# Assessing potential spatial accessibility of health services in rural China: a case study of Donghai county

**DOI:** 10.1186/1475-9276-12-35

**Published:** 2013-05-20

**Authors:** Ruishan Hu, Suocheng Dong, Yonghong Zhao, Hao Hu, Zehong Li

**Affiliations:** 1 Institute of Geographic Sciences and Natural Resources Research, Chinese Academy of Sciences, Beijing 100101, China; 2 Graduate University of Chinese Academy of Sciences, Beijing 100049, China; 3 School of Tourism & Research Institute of Human Geography, Xi’an International Studies University, Xi’an 710128, China; 4 School of Geography, Beijing Normal University, Beijing 100875, China

**Keywords:** Potential spatial accessibility of health services, E2SFCA, Shortest travel times, Villages, Donghai county, China

## Abstract

**Introduction:**

There is a great health services disparity between urban and rural areas in China. The percentage of people who are unable to access health services due to long travel times increases. This paper takes Donghai County as the study unit to analyse areas with physician shortages and characteristics of the potential spatial accessibility of health services. We analyse how the unequal health services resources distribution and the New Cooperative Medical Scheme affect the potential spatial accessibility of health services in Donghai County. We also give some advice on how to alleviate the unequal spatial accessibility of health services in areas that are more remote and isolated.

**Methods:**

The shortest traffic times of from hospitals to villages are calculated with an O-D matrix of GIS extension model. This paper applies an enhanced two-step floating catchment area (E2SFCA) method to study the spatial accessibility of health services and to determine areas with physician shortages in Donghai County. The sensitivity of the E2SFCA for assessing variation in the spatial accessibility of health services is checked using different impedance coefficient values^a^. Geostatistical Analyst model and spatial analyst method is used to analyse the spatial pattern and the edge effect of potential spatial accessibility of health services.

**Results:**

The results show that 69% of villages have access to lower potential spatial accessibility of health services than the average for Donghai County, and 79% of the village scores are lower than the average for Jiangsu Province. The potential spatial accessibility of health services diminishes greatly from the centre of the county to outlying areas. Using a smaller impedance coefficient leads to greater disparity among the villages. The spatial accessibility of health services is greater along highway in the county.

**Conclusions:**

Most of villages are in underserved health services areas. An unequal distribution of health service resources and the reimbursement policies of the New Cooperative Medical Scheme have led to an edge effect regarding spatial accessibility of health services in Donghai County, whereby people living on the edge of the county have less access to health services. Comprehensive measures should be considered to alleviate the unequal spatial accessibility of health services in areas that are more remote and isolated.

## Introduction

The impact of geographical location on health is increasingly examined [1,2]. Although definitions of “rural” are diverse, both developed and developing counties are characterized by large gaps in accessibility between the health services in rural versus urban areas [3]. China’s rural areas are no exception. Although China’s economy has grown rapidly in recent years, the percent of GDP spent on health care has failed to increase, and most health resources have been concentrated in urban areas [4-6]. During the 1990s, only 20% of the government’s public health spending was used on the rural health system that served 70% of the Chinese population [4]. According to the National Bureau of Statistics of China, the rural population stood at 713 million at the end of 2009, constituting 53.4 percent of the total population, which was more than 1.33 billion. The ratio of medical technical personnel in urban versus rural areas was 4.26:1 in 2009 [6]. The rural Chinese population is characterized by poorer health status and limited access to appropriate health services compared with those of urban dwellers. In China, life expectancy rates are lower in rural areas than they are in urban areas [7]. The newborn mortality rates and maternal mortality rates in rural areas are, respectively, 2.4 times and 1.28 times the rates of urban areas [6]. Many factors, including insufficient access to health services and insufficient income, may lead to the lower health status in rural areas. Increases in income and the New Cooperative Medical Scheme (NCMS) pilot program are reducing financial factors that limit access to health services in rural China, while the geographical location of rural citizens is playing an increasingly important role. According to the POVERTY MONITORING REPORT OF RURAL CHINA 2010, the percentage of rural Chinese citizens who were unable to receive timely health services for financial reasons dropped by 7.8% from 2002 to 2009. However, the percentage of people who were unable to access health services due to long travel times and transportation costs increased by 10.1% from 2002 to 2009 [8]. Eliminating disparities in spatial accessibility of health services and improving the health of the population should receive greater consideration by the Chinese public health care system. The first step entails careful government agency planning and identifying the truly underserved populations [9-11]. It is necessary for academics and policy makers to use reliable and robust measures to determine variation in the spatial accessibility of health services [2,12]. Here, we take Donghai County in China as the study unit to analyse areas with physician shortages and characteristics of the potential spatial accessibility of health service at the county level. This paper mainly analyses how the health services resources distribution and the New Cooperative Medical Scheme affect the potential spatial accessibility of health services in Donghai County, and we also give some advice on how to reduce the unequal spatial accessibility of health services in areas that are more remote and isolated. We hope that this analysis will help public health care policy makers in a degree.

As a measure for determining areas with insufficient health services, spatial accessibility of health services refers to the relative access to health services in a given location [13,14]. Spatial accessibility of health services is influenced primarily by travel distance or travel time and the spatial distribution of health service providers and consumers [13]. The spatial accessibility of health services can be classified into two main categories, potential and revealed spatial accessibility, based on the actual use of health services [15-18]. Revealed spatial accessibility of health services refers to the actual use of health care services in a given location, whereas potential spatial accessibility of health services refers to the aggregate health service resources that are available in an area. Potential access is the basis for estimating revealed access. In this paper, we will focus on measuring potential spatial accessibility.

### Methodology and procedures

In China, the rural and urban health care systems differ [19]. Urban residents are covered by a compulsory employment-based basic medical insurance scheme and an urban resident scheme. For rural areas, the central government of China launched the NCMS in 2003. The NCMS is a voluntary health insurance scheme designed to relieve the excessive financial burden of health services in rural areas [20]. The NCSM is considered a primary medical security system in rural China, primarily providing financial protection against catastrophic illness [21]. The risk pooling unit under NCMS is the county, and every county has its own reimbursement scheme. The NCMS coverage rate in Donghai County is over 98% in all villages. The reimbursement rates within the county border and outside the county border differ. For inpatient services received outside the administrative boundary, the reimbursement rate is 50%, lower than that for those received within the county. For outpatient services received outside the county, there is no reimbursement, whereas the reimbursement rate is 35% for outpatient services received within the county. Individuals who request health services outside the county boundary have greater out-of-pocket payments. In Donghai County, the average inpatient cost per capita was $590.20 US in 2010. For inpatient services received outside Donghai County, patients paid an additional $147.50 US. This reimbursement scheme affects this paper’s analysis. In some study cases, researcher can provide health services to people at the edge of the study area, where there are fewer hospitals, by extending the boundary with the buffer zone [22]. We assume that people cannot obtain health services outside the county due to the NCMS reimbursement scheme in Donghai County.

The enhanced two-step floating catchment area (E2SFCA) method applied in this paper was first proposed by Luo and Qi [11]. The method originated from the two-step floating catchment area (2SFCA) method proposed by Radke and Mu [23] optimised by Wang and Luo [10]. The 2SFCA method is a commonly employed special case of the gravity model that intuitively interprets accessibility [10]. The shortcoming of the 2SFCA method is that it assumes that access does not diminish with distance within a catchment area. When it’s used in a large catchment area, however, the assumption is not appropriate. Thus, Luo and Qi introduced weights for different travel time zones within a catchment area to account for distance decay, and their method is referred to as the “E2SFCA method” in this paper [11]. The E2SFCA method combines the population-to-provider ratio, distance or time to nearest services and gravity models into one framework [24,25]. The population-to-provider ratio is commonly used because it is easy to calculate the ratio using standard boundaries, and these ratios are intuitive and readily understood [1,13]. The population-to-provider ratio is traditionally used to assess the spatial accessibility of health services for Donghai County’s statistical yearbook. The shortcoming of this method is the difficulty in revealing detailed spatial variations within large areas [26]. The use of travel distance or time to the nearest service solves the problem of proximity but it neither accounts for competition among providers and consumers nor effectively measures spatial accessibility when there is more than one health service to choose from [26-28]. The gravity model assumes that spatial accessibility diminishes with increased distance and addresses the shortcomings of the travel impedance method by integrating both proximity and availability [9,26,29]. Criticisms of the gravity model have focused on the difficulty in obtaining needed traffic data and calculating the distance-decay function [11]. Due to a lack of data, this problem remains unresolved in some applications, and empirical value is often an acceptable substitute [30,31]. The E2SFCA and 2SFCA methods differ because E2SFCA differentiates accessibility within a catchment area, and multiple travel time zones within each catchment area are assigned different weights according to Gaussian function [31]. Using the E2SFCA method, spatial accessibility of health services is calculated as follows:

For the first step, assign an initial ratio to each hospital centered at a village as a measure of service access: Define the catchment area of health services location *j* as the area within a 30-min driving zone, an acceptable catchment size for primary health services [31]. Break the 30-min time zone into three travel time zones based on ranges of 0–10, 10–20 and 20–30 min. Assign three 10-min time zones with different distance weight *Wr*. The *Wr* is calculated from a Gaussian function which means that the access to physician diminishes with distance within the 30-min time zone. The function is calculated as follows [31]:

(1) $$\mathrm{Wr}=f\left(d_{\mathrm{ij}}\right)=f\left(d_{\mathrm{kj}}\right)=f\left(z\right)=\exp\left(-{\left(z-1\right)}^{2}/\beta \right)$$

Where *Wr* is distance weight, *d*_*ij*_ is the distance from hospital to village, *d*_*kj*_ is the distance from village to hospital, *β* is the impedance coefficient [1] and z is the zone number and

(2) $$z=\left\{\begin{array}{c} 1\;,\begin{array}{ccc} \mathrm{if}\;0<d_{\mathrm{kj}}\leq \frac{c_{j}}{3} & \mathrm{or} & \mathrm{if}\;0<d_{\mathrm{ij}}\leq \frac{c_{i}}{3} \end{array}\\ 2\;,\begin{array}{ccc} \mathrm{if}\frac{c_{i}}{3}<d_{\mathrm{kj}}\leq \frac{2c_{j}}{3} & \mathrm{or} & \mathrm{if}\frac{c_{i}}{3}<d_{\mathrm{ij}}\leq \frac{2c_{i}}{3} \end{array}\\ 3\;,\begin{array}{ccc} \mathrm{if}\frac{2c_{i}}{3}<d_{\mathrm{kj}}\leq \;c_{j} & \mathrm{or} & \mathrm{if}\frac{2c_{i}}{3}<d_{\mathrm{ij}}\leq \;c_{i} \end{array} \end{array}\right)$$

Where c is the catchment size (30 m driving time) and the other variables are the same as those in equation 1. Here, we used *β* = 1.5 and *β* = 2.0 to obtain the weights to calculate the spatial accessibility values and text the variation of spatial accessibility sensibility. The *β* value used here is an empirical value that Peters and Thomas calculated [32].

(3) $$\begin{array}{cc} \begin{array}{ccc} w_{\mathrm{kj}} & \mathrm{or} & w_{\mathrm{ij}} \end{array}= & \left\{\begin{array}{c} 1\;,\begin{array}{ccc} \mathrm{if}\begin{array}{cccc} d_{\mathrm{kj}} & \mathrm{or} & d_{\mathrm{ij}} & \in \mathrm{zone}1 \end{array} & & \end{array}\\ \;0.51\;,\begin{array}{ccc} \mathrm{if}\begin{array}{cccc} d_{\mathrm{kj}} & \mathrm{or} & d_{\mathrm{ij}} & \in \mathrm{zone}2 \end{array} & & \end{array}\\ \;0.07\;,\begin{array}{ccc} \mathrm{if}\begin{array}{cccc} d_{\mathrm{kj}} & \mathrm{or} & d_{\mathrm{ij}} & \in \mathrm{zone}3 \end{array} & & \end{array} \end{array}\right)\\ & \begin{array}{cc} \mathrm{if} & \beta =1.5 \end{array} \end{array}$$

(4) $$\begin{array}{cc} \begin{array}{ccc} w_{\mathrm{kj}} & \mathrm{or} & w_{\mathrm{ij}} \end{array}= & \left\{\begin{array}{c} 1\;,\begin{array}{ccc} \mathrm{if}\begin{array}{cccc} d_{\mathrm{kj}} & \mathrm{or} & d_{\mathrm{ij}} & \in \mathrm{zone}1 \end{array} & & \end{array}\\ \;0.61\;,\begin{array}{ccc} \mathrm{if}\begin{array}{cccc} d_{\mathrm{kj}} & \mathrm{or} & d_{\mathrm{ij}} & \in \mathrm{zone}2 \end{array} & & \end{array}\\ \;0.14\;,\begin{array}{ccc} \mathrm{if}\begin{array}{cccc} d_{\mathrm{kj}} & \mathrm{or} & d_{\mathrm{ij}} & \in \mathrm{zone}3 \end{array} & & \end{array} \end{array}\right)\\ & \begin{array}{cc} \mathrm{if} & \beta =2.0 \end{array} \end{array}$$

Search all population locations (*k*) that are within a threshold travel time zone (*Dr*) from hospital *j* and compute the weighted physician-to-population ratio within the catchment area as follows:

(5) $$\begin{array}{l} R_{j}=\frac{S_{j}}{\sum_{K\in \left\{d_{\mathrm{kj}}\in D_{r}\right\}}P_{k}W_{r}}\\ \;=\frac{S_{j}}{\sum_{K\in \left\{d_{\mathrm{kj}}\in D_{1}\right\}}P_{k}W_{1}+\sum_{K\in \left\{d_{\mathrm{kj}}\in D_{2}\right\}}P_{k}W_{2}+\sum_{K\in \left\{d_{\mathrm{kj}}\in D_{3}\right\}}P_{k}W_{3}} \end{array}$$

Where *d*_*kj*_ is the traveling time between hospital *j* and administrative village *k*, and *P*_*k*_ is the population of the administrative village *k* that falls within catchment area size *j* (*d*_*kj*_∈*Dr*). *S*_*j*_ is the number of hospital staff at location *j*. *D*_*r*_ is the *rth* travel time zone (r = 1-3) within the catchment area. *Wr* denotes a predefined distance-weight for *Dr*.

For the second step, sum the initial ratios in the overlapped hospital areas to measure access for a village where rural residents have access to multiple hospitals. The procedure is: For each administrative village location *i*, search all hospitals *j* that are within the threshold of 30 minutes’ travel time zone from administrative village *i*, and sum the supply-to-demand ratios *R*_*j*_ at those villages to obtain accessibility *A*^*F*^_*i*_ as follows:

(6) $$\begin{array}{l} A_{i}^{F}=\sum_{{}^{j\in }\left\{d_{\mathrm{ij}}\in \;D_{r}\right\}}R_{j}W_{r}\\ \;=\sum_{{}^{j\in }\left\{d_{\mathrm{ij}}\in \;D_{1}\right\}}R_{j}W_{1}+\sum_{{}^{j\in }\left\{d_{\mathrm{ij}}\in \;D_{2}\right\}}R_{j}W_{2}+\sum_{{}^{j\in }\left\{d_{\mathrm{ij}}\in \;D_{3}\right\}}R_{j}W_{3} \end{array}$$

Where *A*^*F*^_*i*_ is the accessibility of the village at location *i* to the hospital j; *d*_*ij*_ is the travel time between *i* and *j*; and *R*_*j*_ is the doctor-to-population ratio at hospital *j* that falls within the catchment area centered at *i* (that is, *d*_*kj*_∈*Dr*). *Wr* is distance weight *f* (*d*_*ij*_) (see also equation 1). A larger value of *A*^*F*^_*i*_ indicates greater access for a village.

The E2SFCA method can be implemented in ArcGIS 9.3 by the procedures using a series of “join” operations, the “sum” and “field calculation” operations are used in the 2SFCA method [14,33].

### Study area and data

**Figure 1:**
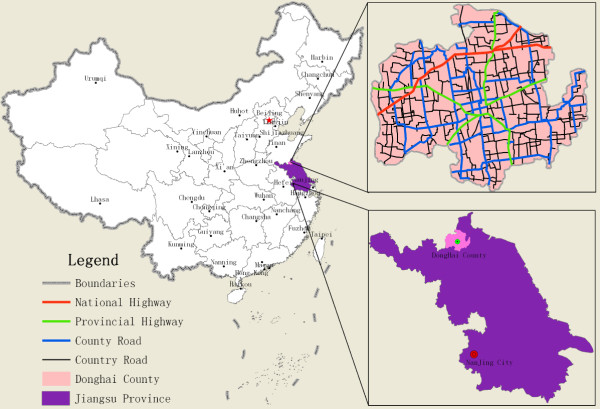
Position and Traffic Network of Donghai County Jiangsu Province, China.

In this paper, we choose Donghai County of Jiangsu Province, China as the study area. Donghai County is a provincial-level poor county (the average village income per year is 1020 US$ as of 2009) located in the northeast of Jiangsu Province. Its latitude and longitude are 34°11′-34°44′N and 118°23′-119°10′E, respectively (See Figure 1). There are 347 administrative villages, and they have a total population of 977,984, approximately 86.5% of the entire population of the county. The 2009 demographic and economic data of the administrative zones were provided by the Statistics Bureau of Donghai County.

The rural health care system in Donghai County consists of a three-tier health care network of county hospitals, township hospitals, and village clinics. Data regarding the number of hospital personnel are provided by the Health Bureau of Donghai County. In Donghai County, there are 535 medical institutions, including 2 county-level hospitals, 25 town-level hospitals, and 508 clinics. Health services in village clinics are at a very low level. Of the 508 clinics, 355 village clinics, accounting for 69.3%, have no assistant doctor or high-level doctor, and 67 village and private clinics, accounting for 19%, have one assistant doctor. The remaining 12% have one or more assistant or high-level doctors. The health services of village clinics include treatment for common diseases, reports of infectious disease epidemics, immunisation, and maternal and child health services. To compare the health services in the county, 27 town-level or county-level hospitals, each equipped with more than 20 beds, and a total 2,371 health personnel are selected as the health service providers. These selected hospitals can accommodate general surgery and provide other primary health care. Donghai County People’s Hospital, located in the county seat (Niushan Town), provides the best health services among all the hospitals in Donghai County. This hospital, with approximately 30% of the total health service personnel of the selected hospitals, is the only hospital that can perform CT examinations and address unusual or more complex health problems.

To enable the hospitals’ practice locations to be used within the Geographical Information System (GIS), the locations are digitized as latitude and longitude points based on the Donghai County communication map. We use the road network to compute the shortest travel times from the villages to the hospitals. The road network in this study area is also digitized according to the Donghai County communication map. Based on road usability, road class and 2004 Chinese highway technical standards, the standard speed limit is 60 km/h at the national and provincial levels, 40 km/h at the county and town levels and 20 km/h for the village road network. We assume that village residents would travel by motorcycle. Since every family does not own a car, we assume that they use public transport or rented cars. The shortest travel times from the hospitals to the administrative villages and the times from the villages to the nearest hospitals can all be calculated using the Origin–destination (OD) cost matrix function in the Network Analyst Extension and the field calculator of ArcGIS 9.3 [14,33].

## Results

Calculations of the shortest travel time from the villages to the hospitals show that 64% of the villages are living beyond 30 minutes’ journey to the nearest hospital and 10% are beyond 60 minutes, 36% are within 30 minutes and 9% are within 15 minutes.

**Figure 2:**
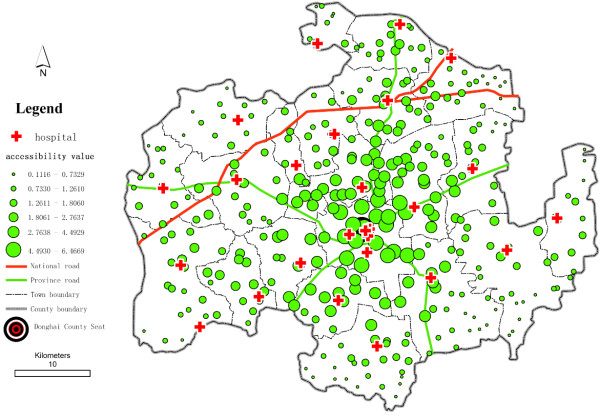
**Potential spatial accessibility of health services in Donghai County, China, using the E2SFCA method (*β *** **=1.5).**

**Figure 3:**
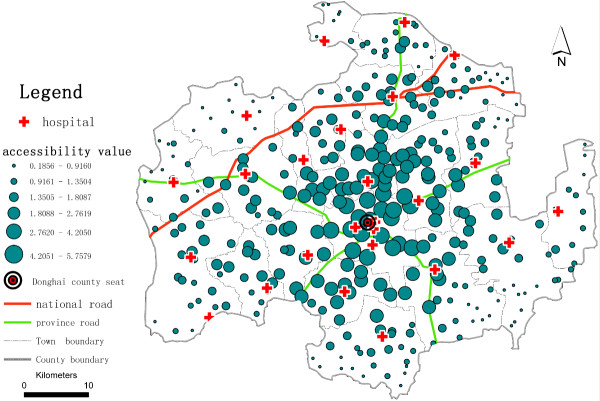
**Potential spatial accessibility of health services in Donghai County, China, using the E2SFCA Method (*β *** **=2.0).**

To determine the potential spatial accessibility of health services, we take the ratio of health professionals per 1,000 residents as the accessibility score. In China, there are no national standards for health service access to use as basic indicators to determine areas with physician shortages; consequently, we use average levels as a reference to define underserved health service areas. Here, we compare the values of spatial accessibility of health services among the villages, compute for the catchment area size of 30 m, and use *β* = 1.5 and *β* =2.0 (see Table 1). We find that a substantial gap exists between the higher and lower potential spatial accessibility of health services. A higher *β* value (e.g., 2.0) yields a higher average accessibility score with low standard deviation. When we use *β* =2.0, 67% of villages have accessibility scores lower than the average level; 79% of village accessibility scores are lower than the province average level (3.065 health professionals per 1,000 residents in 2009). Lower *β* value (e.g., 1.5) yields greater variation in potential spatial accessibility of health services. With *β* =1.5, we find that 69% of villages have potential spatial accessibility of health services lower than the average for Donghai County, and 79% of the village scores are lower than the average for Jiangsu Province. We compare the spatial variation of the potential spatial accessibility of health services (Figure 2 and Figure 3). As shown in Figure 2 and Figure 3, villages with the highest potential spatial accessibility of health services cluster around the county seat (Niushan Town), where the intersection of a national road and a provincial road provides convenient roadway access. This concentric spatial pattern exists because the best health service resources are distributed throughout the county seat. The county seat is an administrative centre, and in addition to Donghai County People’s Hospital, there are six other town-level hospitals in the area. The health personnel of these 7 hospitals constitute approximately 48.4% of the total personnel of the selected hospitals. In addition to the area around the county seat, villages located near national or provincial roads, which have higher speed limits, have higher potential spatial accessibility of health services. The potential spatial accessibility scores of health services are lower for villages in the outlying areas of the county than those in the centre of the county. There are at least two reasons why villages in the outlying areas of Donghai County have the lowest potential spatial accessibility of health services. First, the villages are sparsely populated. Second, people living in the outlying areas have fewer hospitals to choose from than people living in the centre area. We assume that those villages could not obtain health services outside Donghai County because of the administrative restrictions under the NCMS.

**Table 1:** Comparison of accessibility measures

***β *** **value**	**Minimum**	**Max**	**Average**	**Standard deviation**
1.5	0.11	6.47	1.91	1.31
2.0	0.19	5.76	1.95	1.23

**Figure 4:**
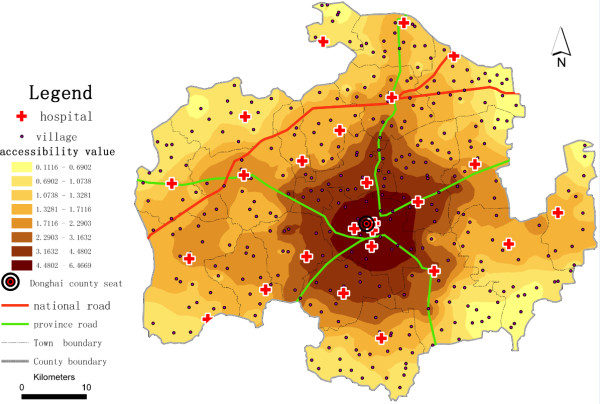
**IDG Interpolation of potential spatial accessibility of health services in Donghai County, China (*β*** **= 1.5).**

**Figure 5:**
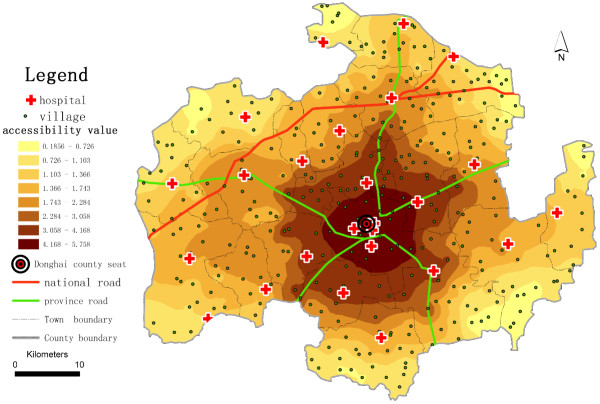
**IDG Interpolation of the potential spatial accessibility of health services in Donghai County, China (*β*** **= 2.0).**

**Figure 6:**
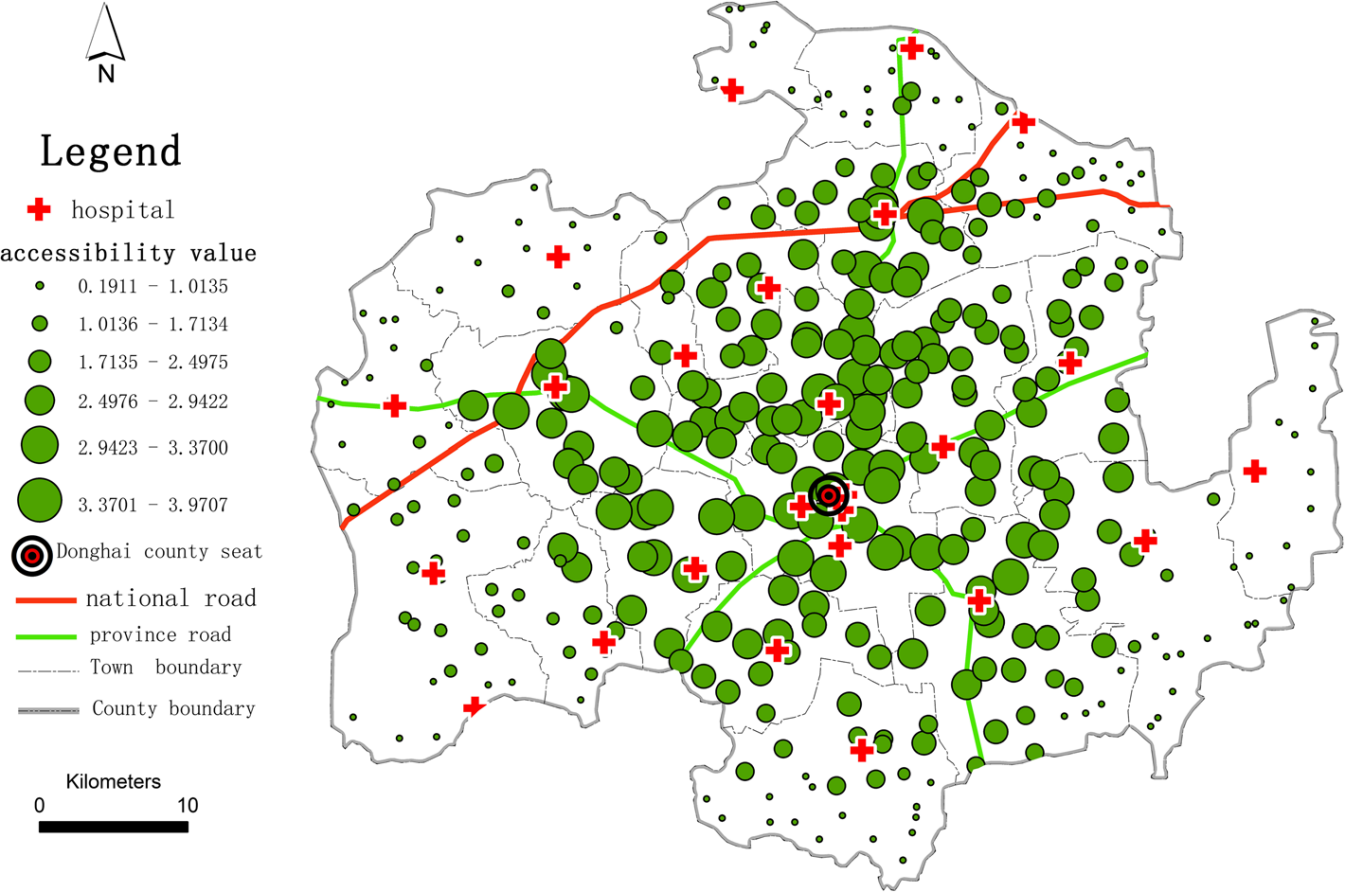
**Access to health services in Donghai County, China using the 2SFCA Method (*Dr = 30 m*).**

To further assess the continuous changes of the potential spatial accessibility of health services in Donghai County, we use inverse distance weighting (IDW) interpolation^b^, a method of geostatistical models, on the spatial accessibility scores of the villages (see Figure 4 and Figure 5) to generate potential spatial accessibility values around the village points and to address the problem that we encounter when we extract the village as a point. Using a higher *β* value reduces the gap in potential spatial accessibility of health services between adjacent villages compared with using a lower *β* value, and yields a smoother shape of IDW interpolation. The lower *β* value is more suitable compared with using a higher *β* value in this study case. It makes the potential spatial accessibility of health services diminish clearly with the increase of travel distance. We also compare the results of the E2SFCA and 2SFCA methods (see Figure 6). The results strongly demonstrate that villages near the county seat receive lower accessibility values with the 2SFCA method than with E2SFCA, whereas the average access value of all villages is higher than the values that the E2SFCA method calculates.

## Discussion and conclusion

In this paper, we analyse the spatial accessibility of health services, and we do not compare interactions between spatial and non-spatial factors of health service accessibility. Calculations of health service users still can reflect the influence of social factors, such as cost of care, staff training and skill levels, laboratory capabilities, pharmaceutical supplies in health facilities and work responsibilities. Because detailed patient data in all hospitals are not available, we use two hospital patient samples as an example: Donghai County People’s Hospital and Huangchuan Town hospital. The latter is located at the edge of Donghai County. The former is equipped with professional physicians and good medical applications. The inpatient and outpatient numbers for Donghai County People’s Hospital are 33,355 and 333,902, respectively, and we calculate 453,984 potential health service users. The inpatient and outpatient numbers for Huangchuan Town hospital (a town-level hospital) are 1,128 and 89,800, respectively, and we calculate 129,716 potential users. The results further suggest that remoter village clinics and town hospitals lack high quality health services, which is a topic worthy of future investigations. In addition to town-level hospitals and county-level hospitals, community and village clinics are located around the county seat. These clinics have no urine and blood examination instruments or fluoroscopy testing equipment. Some clinics have no physician assistants, and folk doctors play the role of the physician assistants. The reimbursement rate for outpatients is higher for community or village clinics than for Donghai County People’s Hospital (30% vs 20%), and the health services price is lower. When rural residents around the county seat perceive their illness to be less severe based on their own experiences, they may seek health services at community or village clinics.

We do not differentiate between individuals with or without vehicles. Most of the rural population in Donghai County do not have private vehicles and rely on public transportation to access health services. It is difficult for village residents to receive timely access to vehicles, even if they can afford the vehicle rental fee. This limitation may lead to an overestimation of the potential spatial accessibility of health services.

The problem of calculating the appropriate impedance coefficient is still insufficiently resolved due to the lack of necessary data and complex computations. These social factors and the problem of *β* may make the potential spatial accessibility of health services unstable but do not significantly change the results of our analysis. Compared with previous studies, the impedance coefficient values have a greater effect on accessibility in our case study (see Table 1). Similar to the impedance coefficient, the 30-minute catchment size is a somewhat arbitrary yet empirically acceptable value in this study. The catchment size is not suitable for areas of low population density in western China. Donghai County is only used as a case study. Most terrain gently slopes in Donghai County, and the road network is better than that in poorer counties in the western countryside of China. The villages in the western counties are more sparsely populated, and the populations live in poverty; moreover, health service resources are concentrated in the county seats. When we assess the potential spatial accessibility of health services using the E2SFCA method, the catchment size and *β* values should be reconsidered.

**Figure 7:**
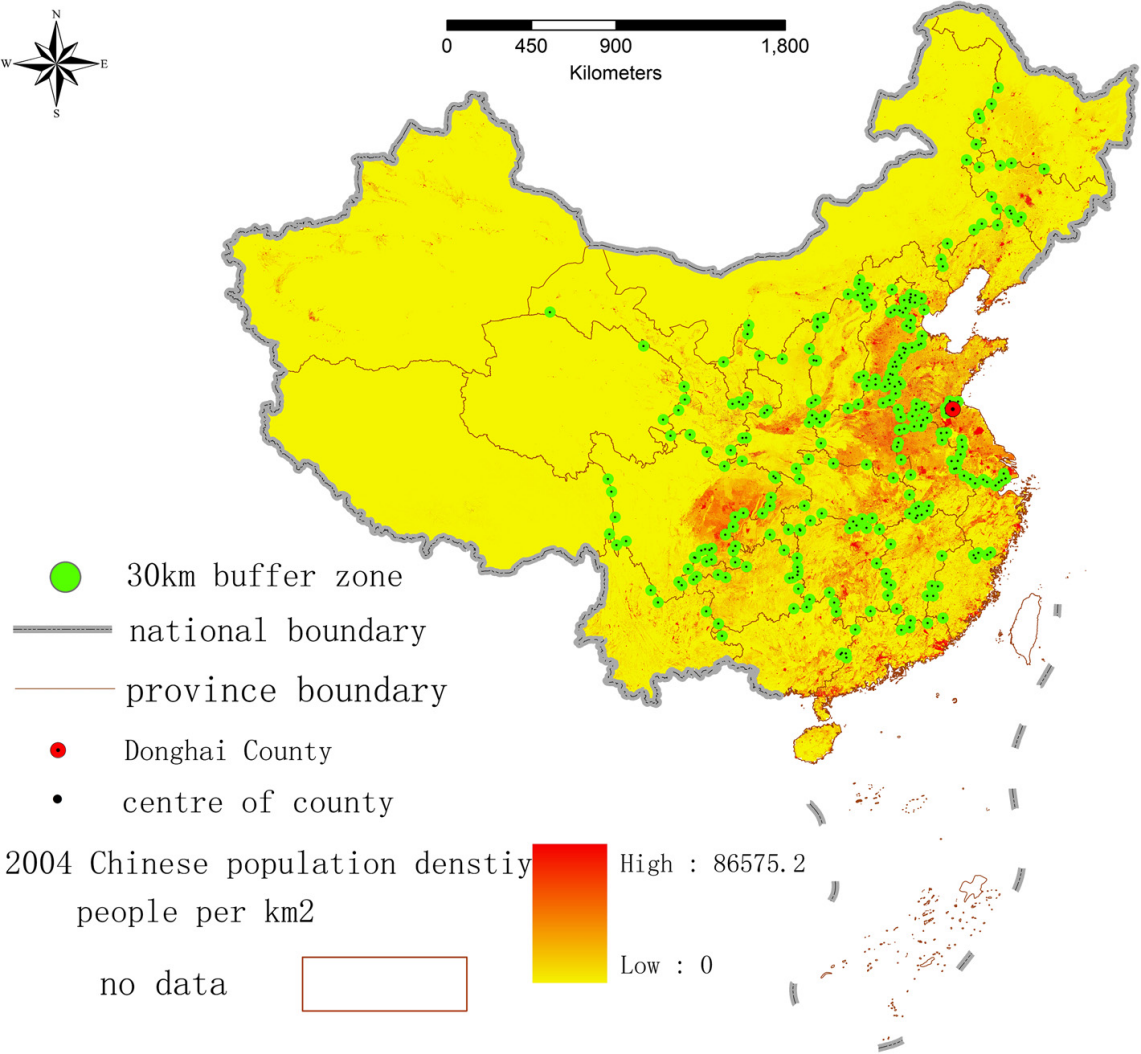
Counties at the Provincial Boundary 15 km Buffer Zone, China.

The administrative restrictions of the NCMS partially led to an edge effect regarding the potential spatial accessibility of health services, whereby areas on the edge of the county have less access to health services. This situation may increase travel costs for health services and decrease the efficiency of health services in rural areas. Although the rural populations can obtain reimbursement throughout a province, we cannot account for edge effects. Approximately 271 counties are located in the 15 km buffer zone from the provincial boundaries. To estimate the number of rural residents potentially affected by this edge effect, we extend the 30 km buffer zone at the county centre and calculate the population within the 30 km buffer zone (see Figure 7). This is calculated by using the 30 km buffer zone map and a floating population density map for China for 2004 with the zonal statistics tool in the ARCGIS 9.3 spatial analyst model. The results indicate that there is a population of approximately 200,000,000 within the catchment area (30 km buffer zone) of rural high quality health services located at these county seats. Without the administrative restrictions regarding reimbursement, a conservative estimate would suggest that approximately 30% of the population of 67 million rural people may select the nearest health services across province boundaries.

The primary conclusion discussed below can be drawn from the results of applying the E2SFCA method and the shortest traffic time for Donghai County. Most of the villages are in underserved health care areas because health services resources are overly concentrated in the area surrounding the county seat. We assume that a lack of interactions across boundaries leads to fewer choices for villages regarding health service providers. Scores for the potential spatial accessibility of health services vary greatly from the centre of the region to the outlying areas, and using a larger impedance coefficient value reduces the gap in spatial accessibility of health services between adjacent villages, whereas using a smaller impedance coefficient value leads to greater disparity among the villages. Higher accessibility values occur along the highway. The E2SFCA method reveals much more detailed variation in spatial accessibility than the 2SFCA method, and it is more suitable for analysing potential spatial accessibility of health services than 2SFCA in this study case. In many of China’s rural areas, health services and physicians tend to cluster in county seats and the surrounding areas; thus, the spatial accessibility patterns show similar characteristics [34,35]. Accordingly, rurality may be the factor driving the unequal potential spatial accessibility of health services. To provide equal spatial accessibility of health services, government interventions should target isolated rural and small town areas.

Consequently, comprehensive measures should be considered to alleviate the unequal spatial accessibility of health services in areas that are more remote and isolated. The government should develop the healthcare system to decrease the disparity between rural and urban areas, work towards universal insurance coverage at the national level, or at least increase the inpatient and outpatient reimbursement rates for rural areas. As a public service, governmental provision of rural health services will remain a key issue. The government should upgrade the academic qualifications and professional skills of physicians in village clinics, expand the road networks from villages to town and county hospitals and encourage high level hospitals to assume more responsibility for the rural clinics and practitioners in their catchment area. The government should compensate health service providers who work in remote rural areas. An incentive mechanism should make health service prices reasonable and acceptable to the rural population. With the increase of urban populations, rural areas may become even more sparsely populated, especially in western China, and increases in professionals and facilities may be economically unfeasible and inefficient. Mobile health services and telemedicine services are additional realistic measures. Both efficiency and fairness should be considered for future endeavours.

### Endnotes

^a^The impedance coefficient reflects willingness to access a medical service considering the travel cost. In theory, this coefficient should be calculated using actual physician visit data and health service utilisation surveys using a statistical method. However, these data are generally not available. As a substitute, researchers generally use empirical impedance coefficients to compute potential spatial accessibility of health services.

^b^Inverse distance weighting (IDW) interpolation explicitly relies on the assumption that things that are close to one another are more alike than things that are farther apart. To predict a value for any unmeasured location, IDW uses the measured values surrounding the prediction location. Those measured values closest to the prediction location have more influence on the predicted value than those values farther away. Thus, IDW assumes that each measured point has a local influence that diminishes with distance. IDW puts greater weight on the points closer to the prediction location compared with those farther away, hence the name inverse distance weighting. This method can address the shortcoming of extracting the villages as points.

## Competing interests

The authors declare that they have no competing interests.

## Authors’ contributions

All authors contributed to the design of the study. RH, HH and ZL collected and analysed the research data. RH and HH drafted the manuscript. RH, SD and YZ revised the paper. All authors read and approved the final manuscript.
